# Histoplasmosis laryngeal: report first case in Colombia

**Published:** 2014-12-30

**Authors:** Carlos Alberto Moriones Robayo, Claudia Patricia Guerra Ortiz

**Affiliations:** 1 Médico especialista en Otorrinolaringología. Hospital Universitario del Valle, Clínica Fundación Valle de Lili, Colombia. Facultad de Salud, Universidad del Valle. Cali, Colombia.; 2 Médico especialista en Otorrinolaringología. Universidad del Valle.

**Keywords:** Histoplasmosis, granulomatous inflammation, supraglottic mass

## Abstract

Laryngeal histoplasmosis is a fungal infection that is frequent in Colombia. Laryngeal histoplasmosis usually occurs in immunocompromised patients through the dissemination of the fungus from the lungs to other organs. Histoplasmosis isolated laryngeal (primary) is rare. If a patient presents with a history of immunosuppression by renal transplant, primary laryngeal histoplasmosis with supraglottic granulomatous inflammation that was treated with amphotericin B and Itraconazole, with complete resolution of laryngeal lesions.

## Abstract (es)

Histoplasmosis es una infección fúngica que es frecuente en Colombia. La histoplasmosis laríngea por lo general se presenta en pacientes inmunocomprometidos por la difusión del hongo desde los pulmones a otros órganos. La enfermedad laríngea aislada (primaria) es rara. Se presenta el caso de un paciente con antecedente de inmunosupresión por trasplante renal, con histoplasmosis laríngea primaria que produjo inflamación granulomatosa supraglótica, tratado con Anfotericina B e Itraconazol, con resolución completa de las lesiones laríngeas.

## Introduction

 Laryngeal histoplasmosis is a fungal infection that is frequent in Colombia, especially in the department of Antioquia where have been reported until 2008 the highest number of cases followed by Valle del Cauca and Cundinamarca although there are cases reported in 20 of the 32 departments 1. Laryngeal histoplasmosis usually occurs in patients immunocompromised by the spread of the fungus from the lungs to other organs. Isolated laryngeal disease (primary) is rare. We show the case of a patient with immunosuppression for kidney transplant, with primary supraglottic laryngeal histoplasmosis which produced granulomatous inflammation, treated with amphotericin and Itraconazole with complete resolution of laryngeal lesions. The laryngeal histoplasmosis usually occurs in immunocompromised patients by spreading the fungus from the lungs to other organs. Isolated laryngeal histoplasmosis (primary) is so rare.

## Case report

**Figure 1.**
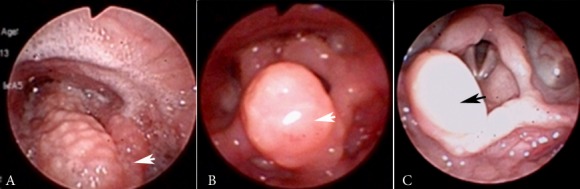
Fiber optic Laryngoscope. Black Arrow: epiglottis. Granulomatous supraglottic edema and deformity of epiglottis (A). Control at 46 days (B) and 8 months (C) starting antifungal therapy.

**Figure 2.**
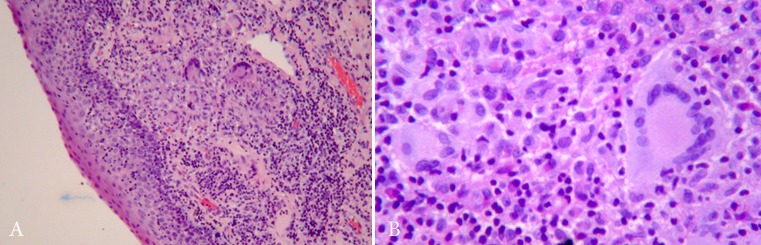
Epiglottis Biopsy, HE staining. A: Injury lined by respiratory epithelium with abundant mixed inflammatory infiltrate, histiocytes, plasma cells, polymorphonuclear cells, and multinucleated giant cells. Without necrosis or vasculitis. (x100) B: mixed infiltrate and multinucleated giant cells (x400).

 Patient male age 7 years old, from Riosucio (Caldas, Colombia), who consults with clinical picture of three weeks duration of diarrhea, odynophagia, hiporexia, headache, occasional fever, stridor during sleep, osteomialgias and decay. Presents a history of renal transplant three years ago, bilateral congenital oligomeganefronia with secondary chronic renal failure. Receiving medications such as Tacrolimus, Mycophenolate and Diltiazem. Physical examination shown erythematous tonsils with mild hypertrophy, stridor and cervical lymphadenopathy. Paraclinical reported a blood count and neutrophil leukocytes, C-Reactive Protein: 1.6, coprological techniques: normal, negative urine culture, negative PPD test, tests for Epstein Bar virus, cytomegalovirus, *Aspergillus galactomannan* and BK polyomavirus negative. The chest radiograph was found parahiliar congestion without bindings and abdominal ultrasonography showed chronic graft nephropathy with normal characteristics, without further alteration. Fiber Optic Laryngoscope is performed and is found edema of granulomatous aspect of supraglottic mucosa which deforms the epiglottis and partially obstructs the airway (Fig. 1). A tracheostomy tube and subsequently mass biopsy was performed without being found necrosis chronic granulomatous inflammation (Fig. 2). Immunohistochemical study discarded lymphoepithelial lesion. In addition colorations were performed for fungi and mycobacteria were negative (BK, PAS, Gomori). Suspecting fungal infection is requested titles for coccidioidomycosis and immunodiffusion for *Histoplasma*, the latter resulting positive. Primary diagnosis of laryngeal histoplasmosis is made (discarding pulmonary focus). The culture Urine and blood cultures for bacteria and fungi were negative at 42 days of incubation. Amphotericin B is initiated at 0.8 mg/ kg/day, and 10 days is changed to Itraconazole 6 mg/kg orally every 12 h for signs of nephrotoxicity, to continue for 12 months on an outpatient basis. 

 In Fiber Optic Laryngoscope control at 46 days of started the amphotericin B is found granulomatous decreased edema and minimal deformity of the epiglottis. A later check was performed at 8 months observing total resolution of lesions in the larynx (Fig. 1).

## Discussion

The causative agent of histoplasmosis is *Histoplasma capsulatum capsulatum* or *Histoplasma capsulatum duboisii*, dimorphic fungus, thermal and intracellular. It is isolated from nature in temperate zones and tropical humid áreas, where soils are acidic, rich in nitrogen. This fungus can be found in soil contaminated by feces of bats or birds such as caves or houses. The handling of contaminated material makes small spores of *Histoplasma capsulatum* become volatile to be easily transported by wind currents over long distances. Human infection usually inhaled by mycelia, It´s the natural infectious form, which is captured by pulmonary macrophages, inside they germinate giving blastopores. Later in immunocompetent individuals, occurs an inflammation at the site of infection, resulting in formation of a or not caseous. Usually the organism is destroyed more or less time, and infection is resolved or asymptomatic, but in cases of deficient cellular immunity, are released from the phagolysosome passing the cytoplasm, where they multiply freely, and the infection will spread to other body systems. Infection stimulates the proliferation of infected macrophages 2 ^,^ 3 . The factors that influence the progression of primary infection are the number of organisms inhaled, extremes of age, immunosuppression and malnutrition 4 .

The clinical spectrum of the disease is variable and can manifest as a severe multisystem disease affecting bone, liver, spleen, lungs, gastrointestinal tract, skin, adrenal glands, larynx or other extrapulmonary sites. It usually occurs as an acute pneumonia, chronic lung cavity, lung nodules, mediastinal fibrosis and granulomatous mediastinitis 2 ^,^ 5 .

The medical condition of laryngeal histoplasmosis may present with dysphonia odynophagia, dysphagia, dyspnea, fatigue, weight loss and generalized unrest. It can also be seen on some occasion leukoplakia lesions and exophytic masses laryngeal and cervical lymphadenopathy 3 ^,^ 4 ^,^ 6 ^,^ 7.

The differential diagnosis includes other laryngeal granulomatous diseases such as tuberculosis, paracoccidioidomycosis, leishmaniasis, blastomycosis, leprosy, syphilis, or actinomycosis. Also, be differentiated from other conditions such as: gastroesophageal reflux, polychondritis, Wegener's disease, sarcoidosis, amyloidosis, rheumatoid arthritis, lupus, carcinoma, lymphoma or papillomatosis 2 ^,^ 5 .

Cultivation and *Histoplasma *biopsy can be made from smears of ulcers in the oral cavity, sputum, bronchoalveolar secretion, urine, and bone marrow biopsies or from oral masses, laryngeal or other organ suspected infection. Organisms can be detected in the culture medium Sabouraud and may take grow up to 6 weeks. The blood culture should be performed in all suspected cases. Using the lysis-centrifugation technique, the culture is positive in 65% of cases 4 ^,^ 7.

In the biopsy can be observed with hematoxylin-eosin granulomatous tissue, necrosis, infiltration of giant cells, lymphocytes, plasma cells and many macrophages. Be used special stains to identify macrophages and this containing hyphae, such as coloring Gomori methenamine-silver, coloring periodic acid-Schiff (PAS) staining Gridley, silver dyes (Grocott) 4 ^,^ 7

Antigen detection in urine and serum should be performed in all patients with suspected disseminated histoplasmosis in order to achieve maximum sensitivity and rapid diagnosis. The enzyme immunoassay can detect *Histoplasma* antigen in urine in about 75% of immunocompetent patients and 95% of the immunocompromised patients. The antigen can be detected in the serum about 100% of cases. A negative antigen test does not rule disseminated histoplasmosis. The antigen can also be detected in cerebrospinal fluid (CSF) in the majority of patients with central nervous system and respiratory samples obtained by bronchoscopy. False positives have been observed in other fungal infections. The antigen detection tests are useful but only if positive, because its sensitivity is poor.

Serological tests for anti-*Histoplasma *using immunodiffusion (serology) and complement fixation methods are positive in about 70% of immunocompromised and 90% of immunocompetent patients with disseminated infection. Antibody tests may be false negative in immunocompromised patients. In acute cases, the antibodies usually appear during the second month after exposure. Antibodies may remain positive for several years and are not necessarily indicative of active disease. In immunodiffusion test, the results are reported as M or H precipitin or bands. Most patients develop a band of M, and H precipitation band is detectable at less than 20% of cases. The H band is observed more frequently in patients with disseminated infection, chronic cavitary pulmonary histoplasmosis or very severe acute pulmonary histoplasmosis. The M band becomes positive before the H band and persist long time 3 ^.^ 9.

The complement fixation test using antigens of yeast and mycelia forms of *H. capsulatum* is slightly more sensitive than immunodiffusion test. However, immunodiffusion test has greater specificity than complement fixation test. The positive results with the complement fixation test may be due to a persistent antibody response from a previous episode of histoplasmosis, infection by other fungi, or other granulomatous diseases 5.

The role of Polymerase chain reaction (PCR) for diagnosis of histoplasmosis is uncertain. The histoplasmin skin test in endemic areas is of little use because of its high number of false positives and its use is reduced to immunocompromised patients 3 ^.^ 9.

It should exclude the involvement of other parts of the digestive air tract and lungs. With a lung infection in the chest radiograph shown multiple calcifications.

If the patient requires hospitalization, does not improve with azoles treatment, is immunosuppressed or show intolerance to azoles, the treatment of choice is intravenous amphotericin B at 0.7-1.0 mg/kg /day, 2-4 months a total administered dose of 35 mg/kg. However, studies have shown that other oral antifungals are effective as amphotericin B in patients with renal impairment and mild to moderate histoplasmosis. Oral fluconazole is administered in a dose of 200-400 mg once a day. The problem with fluconazole is the development of resistance. Therefore, this agent should be reserved for the treatment of patients who cannot take itraconazole. Oral ketoconazole 200-400 mg is administered once a day, but causes more side effects 3 ^.^ 8. The Itraconazole 200 mg administered twice a day for a year in mild to moderate disease, as suggested in the American guidelines (American society of infectious diseases), good treatment success rate. Surgical resection of obstructive masses can be considered and is generally safe for patients whose symptoms do not improve after one to three months of antifungal therapy 5 ^.^ 10.

## Conclusions

Laryngeal histoplasmosis is a rare phenomenon, so it is often misdiagnosed becomes causing a devastating result for the patient. Is likely one of the reasons of the few reported cases is caused by the difficulty in diagnosis. The otolaryngologist should be aware of the existence of laryngeal histoplasmosis and consider it as one of the differential diagnosis when a patient shows signs of granulomatous inflammation or laryngeal masses, to perform specific diagnostic tests for fungi and provide optimal and timely treatment, avoiding unnecessary aggressive interventions.
